# Debate on the relationship between *Helicobacter pylori* infection and inflammatory bowel disease: a bibliometric analysis

**DOI:** 10.3389/fmicb.2024.1479941

**Published:** 2024-11-06

**Authors:** Ziye Wang, Shiqing Zhao, Xiaotian Zhong, Yi Su, Yahan Song, Jun Li, Yanyan Shi

**Affiliations:** ^1^Research Center of Clinical Epidemiology, Peking University Third Hospital, Beijing, China; ^2^Peking University Health Science Center, Beijing, China; ^3^Library, Peking University Third Hospital, Beijing, China; ^4^Department of Gastroenterology, Peking University Third Hospital, Beijing, China

**Keywords:** inflammatory bowel disease, Crohn’s disease, ulcerative colitis, *H. pylori*, intestinal microbiota, immune modulation

## Abstract

**Background:**

Inflammatory bowel diseases (IBD) are chronic inflammation conditions affecting the gastrointestinal tract. Studies point out an association between *Helicobacter pylori* (*H. pylori*) infection and IBD. This study aims to visually assess the research trends and hotspots in the field of *H. pylori* infection and IBD, review mainstream perspectives in this field, and provide a foundation for future research and treatment.

**Methods:**

We searched the Web of Science Core Collection Database for literature related to *H. pylori* and IBD, using VOS viewer to generate visual charts.

**Results:**

A total of 246 publications were included, with articles being the predominant type of document. A significant increase in the number of publications was observed after 2011. China contributed the most of researches. Keyword clusters revealed that the researches primarily focused on immune mechanism, gut microbiome, diagnosis and treatment of IBD. Time trend results indicated that current researches centered on gut microbiota and immune mechanisms.

**Conclusion:**

*H. pylori* infection may have a protective effect on IBD. The exact mechanisms remain unclear and may involve immunomodulation and changes of gut microbiota. Further researches are necessary for better understanding this relationship and its implications for clinical practice. Further researches and clinical practice should pay attention to this topic.

## 1 Introduction

Inflammatory bowel diseases (IBD), including two main types-Crohn’s disease (CD) and ulcerative colitis (UC) (Singh et al., 2017), are a group of chronic conditions affecting the gastrointestinal tract, which are characterized by episodes of abdominal pain, diarrhea, hematochezia, and weight loss (Chang, 2020). In CD, any segment of the gastrointestinal tract can be affected, and the most affected areas are distal ileum, cecum, perianal region, and colon. In UC, the inflammation is confined exclusively to the colon and rectum. Regarding histology, in CD the intestinal pathologies present submucosal thickening, transmural inflammation, ulceration, and non-caseous granuloma. In UC, the inflammation is limited to the mucosal layer, with recesses and abscesses (James et al., 2014; Mak et al., 2020).

More than six million people suffer from IBD worldwide (Imawana et al., 2020). Once in the 20th century, IBD was predominantly regarded as a disease prevalent in Western nations, particularly in Europe and North America (Molodecky et al., 2012; Kaplan and Windsor, 2021). However, the 21st century has witnessed a notable increase in the onset of IBD worldwide, particularly in developing countries in Asia, including China and India (Kaplan, 2015). IBD can not only influence individuals’ quality of life, but also induce other diseases such as malnutrition or even gastrointestinal cancer (Quaglio et al., 2022; Nadeem et al., 2020). The rising prevalence of IBD imposes an escalating socioeconomic burden (Kaplan, 2015). The pathogenesis of IBD involves a combination of host genetic predisposition (Graham and Xavier, 2020), intestinal flora (Glassner et al., 2020; Guo et al., 2022; Haneishi et al., 2023), environmental factors (Mak et al., 2020; Adolph et al., 2022), and immunological abnormalities (Monteleone et al., 2006), but the specific mechanisms is unclear (Mak et al., 2020; Guo et al., 2022). The intestinal flora is crucial to the homeostasis of the gastrointestinal tract, which contributes to immune tolerance process and the development or differentiation of the local and systemic immune system. It may also protect the host from pathogenic intestinal infections. Thus, the dysbiosis of gut microbiota triggers deregulated immune responses, acting as a significant contributor to the development of IBD (Balani et al., 2023).

*Helicobacter pylori* (*H. pylori*) is a Gram-negative, spiral bacterium that infects the stomach. *H. pylori* infection generally occurs in childhood via person-to-person transmission (Li et al., 2023). It is estimated that approximately 50% of the global population is infected with this bacterium (Ren et al., 2022). *H. pylori* infection is a risk factor for various gastrointestinal diseases, including gastritis, peptic ulcers, and gastric cancer. Additionally, some studies report that *H. pylori* infection can increase the risk of autoimmune diseases (Yang et al., 2023). However, other studies suggest that *H. pylori* infection may confer a protective effect against the development of IBD (Fialho et al., 2019; Castaño-Rodríguez et al., 2017). In addition to hygiene hypothesis using to explain the epidemiological correlation between *H. pylori* infection and IBD (Tanner et al., 2023) another potential explanation involves the alteration of gut microbiota composition resulting from *H. pylori* colonization (Bai et al., 2022; Pellicano et al., 2020). However, contrasting evidence exists, with some studies suggesting that *H. pylori* infection is linked to an increased risk of IBD during childhood (Kong et al., 2023; Man et al., 2008; Zhong et al., 2021; Sayar et al., 2019).

In 1969, Alan Pritchard articulated the concept of bibliometrics as “the application of mathematical and statistical methods to books and other media of communication,” using this approach for literature analysis. Bibliometric analysis relies on various publication parameters (such as countries, institutions, and authors) and integrates mathematical and statistical techniques to quantitatively describe the current status, research hotspots, and research trends (Shi et al., 2022). It can process extensive datasets and assess outputs within specific fields (da Cruz et al., 2022). Our study employs bibliometric analysis to examine data pertaining to *H. pylori* infection and IBD.

## 2 Materials and methods

### 2.1 Data sources and screening strategies

We utilized Web of Science Core collection (WOSCC), the most used database for bibliometric analysis to collect literature. This database encompasses a diverse array of prominent international academic journals in the world (Yang et al., 2022). All studies included were identified in WOSCC on February 20, 2024. We employed MeSH database and free text words to improve retrieval accuracy. The search strategy incorporated the following terms include: TS = (“Inflammatory Bowel Disease” OR “inflammatory Bowel Diseases” OR “Crohn’s Enteritis” OR “Regional Enteritis” OR “Crohn’s Disease” OR “Crohns Disease” OR “Inflammatory Bowel Disease 1” OR “Granulomatous Enteritis” OR “Ileocolitis” OR “Granulomatous Colitis” OR “Terminal Ileitis” OR “Regional Ileitides” OR “Regional Ileitis” OR “Idiopathic Proctocolitis” OR “Ulcerative Colitis” OR “Colitis Gravis”) AND TS = (“*Helicobacter nemestrinae*” OR “*Helicobacter pylori*” OR “*H. pylori*” OR “*Campylobacter pylori*” OR “*Campylobacter pylori* subsp. Pylori” OR “*Campylobacter pylori*dis” OR “Campylobacter” OR “*C. pylori*”).

### 2.2 Screening criteria and results

The inclusion criteria are as follows:

1. The literature pertains to topics of IBD and *H. pylori*, and their relationship.
2. The literature was written in English.

The exclusion criteria as follows:

1. Incomplete, corrected or retracted publication.

The complete text was evaluated by two authors independently according to the inclusion and exclusion criteria.

**Figure 1:**
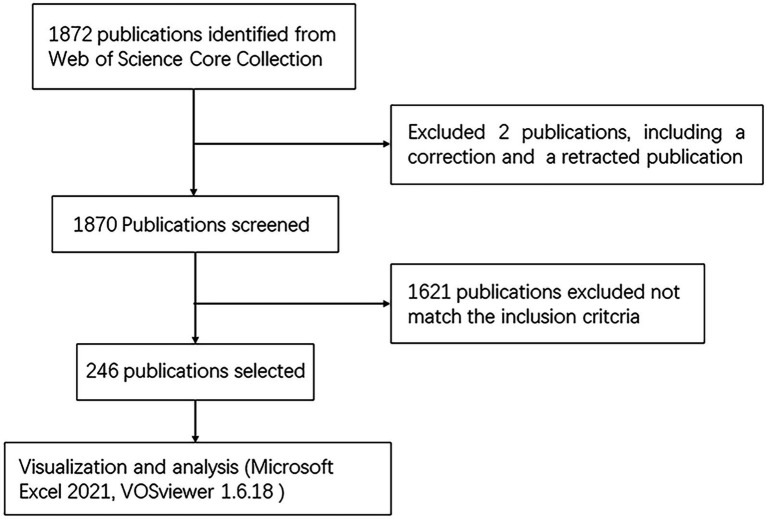
Flowchart of literature selection.

As illustrated in Figure 1, 1,872 articles were identified from the WOSCC database, of which two were excluded due to corrected and retracted. The selection of the remaining papers was conducted by two authors. At last, 246 articles were eventually included.

### 2.3 Data extraction and statistical analysis

Two authors (ZW and SZ) independently evaluated the abstracts and titles of all pertinent publications. Any discrepancy in evaluation was addressed by the third author (YSh). The data from the WOSCC database were collected by Microsoft Office Excel 2021. The software VOSviewer 1.6.18 was used to analyze the data like countries/regions, institutions, authors, keywords, journals, co-authorships, co-occurrences, citations and to generate visual charts (Waltman et al., 2010). In these visualization results, different nodes represent different parameters such as countries/regions, institutions, authors and keywords. Nodes are classified into different clusters according to the degree of linking (distinguished by color). The size of each node refers to the frequency of occurrence of associated parameters and the thickness of the connections between nodes illustrates the strength of linking.

## 3 Results

### 3.1 Publication output

**Figure 2:**
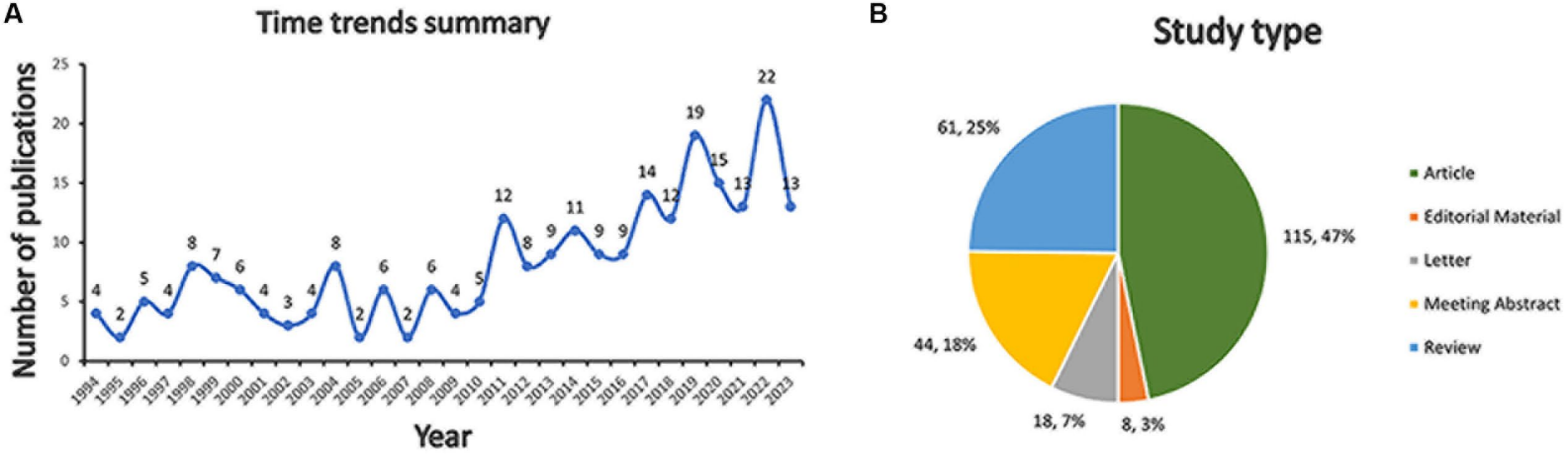
Bibliometric analysis of *H. pylori* and IBD. **(A)** Time trends summary. **(B)** Study type composition.

The 246 documents can be categorized into two periods by time trend of publication, the years 1994 to 2010, and the years 2011 to 2023. The publication counts for each period illustrate the evolving trend of research within this domain (Figure 2A). From 1994 to 2010, a total of 80 publications were recorded. From 2011, the number of publications is increasing, culminating in 22 publications in 2022 alone. From 2011 to 2023, 166 publications were published, accounting for about 67% of the total.

There were 115 (47%) articles, 61 (25%) reviews, 44 (18%) meeting abstracts, 18 (7%) letters, and 8 (3%) editorial materials (Figure 2B).

### 3.2 Country and institution analysis

**Figure 3:**
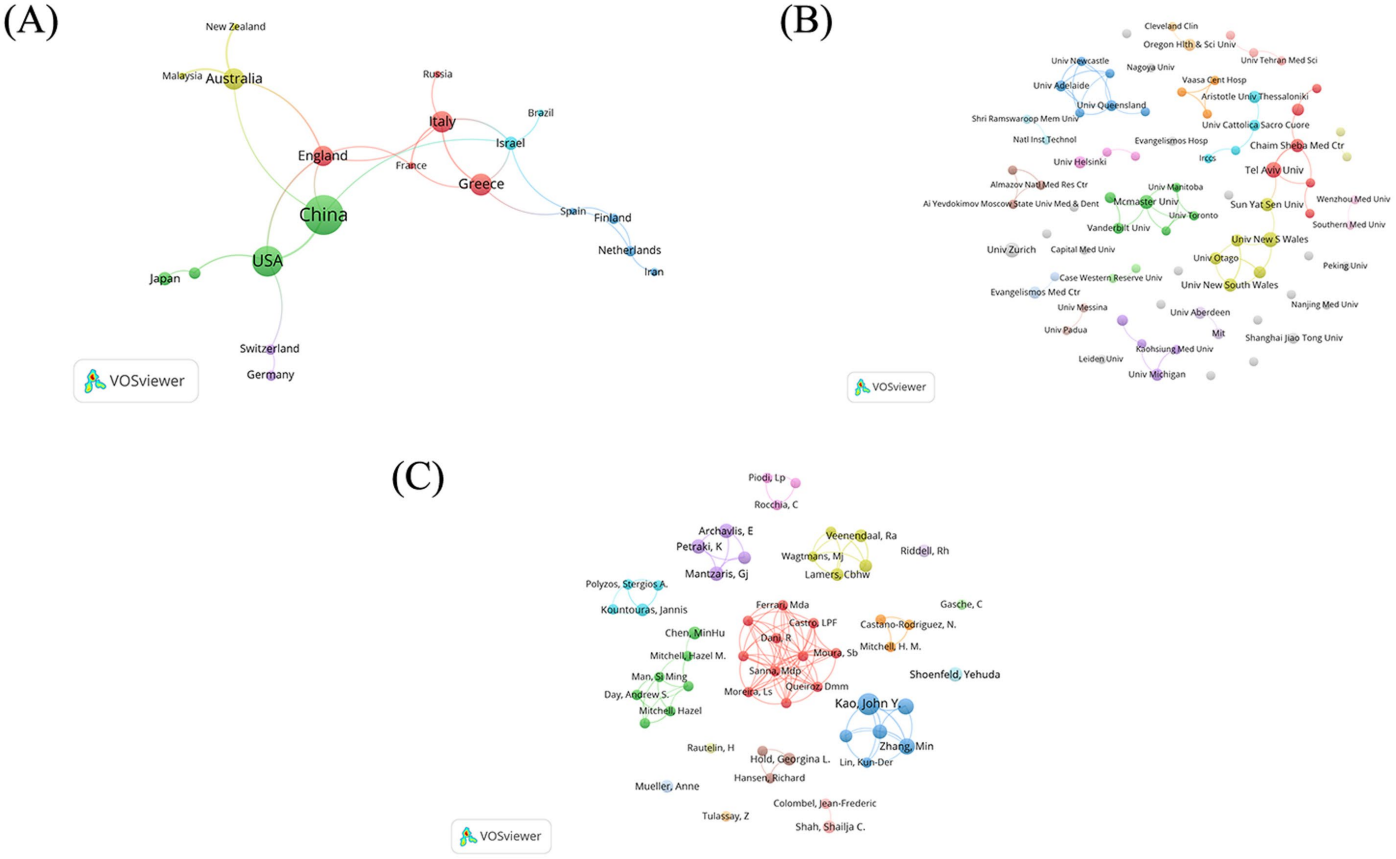
Bibliometric analysis of co-occurrence network map. **(A)** Countries. **(B)** Institutions. **(C)** Authors.

The top 9 countries with the most publications (above 9 records) are listed in Table 1. China published most (42 records), followed by USA (30 records), Greece (20 records) and Australia (19 records). As shown in Figure 3A, China has established cooperative relations with four countries, including Australia, England, USA and Israel. Australia is the most cooperative country with a total link strength (TLS) of 11, collaborating with China, New Zealand, England and Malaysia (Figure 3A).

**Table 1:** Top 9 countries with the most publications.

No.	Countries	Publications	Citations	Total link strength
1	China	42	1,441	7
2	USA	30	813	9
3	Greece	20	671	5
4	Australia	19	646	11
5	Italy	19	861	8
6	England	18	1,286	7
7	Israel	11	605	6
8	Japan	11	150	1
9	Canada	9	236	3

A total of 341 institutions participated in the aforementioned 246 studies (Figure 3B). Among these, 79 institutions published more than 2 documents. The top 9 institutions with the most publications (above 5 records) are detailed in Table 2. Tel Aviv University (Tel Aviv Univ) emerges as the most prolific institution, with 8 related papers, followed by The University of New South Wales (Univ New S Wales) (7 records) in Australia, University of Zurich (Univ Zurich) (7 records) in Switzerland and McMaster University (McMaster Univ) (6 records) in Canada. Tel Aviv University (Tel Aviv Univ) and Chaim Sheba Medical Center (Chaim Sheba Med Ctr) from Israel are the main collaborators (link strength 4). There is a cooperation between Sun Yat-sen University (Sun Yat-sen Univ) in China, the University of New South Wales (Univ New S Wales) in Australia and Tel Aviv University (Tel Aviv Univ) in Israel (link strength 1).

**Table 2:** Top 7 institutions with the most publications.

No.	Institutions	Publications	Citations	Total link strength	Countries
1	Tel Aviv Univ	8	555	555	Israel
2	Univ New S Wales	7	257	257	Australia
3	Univ Zurich	7	286	286	Switzerland
4	Mcmaster Univ	6	195	195	Canada
5	Chaim Sheba Med Ctr	5	487	487	Israel
6	Sun Yat-sen Univ Univ	5	199	199	China
7	Univ New South Wales	5	207	207	Australia

### 3.3 Author and journal analysis

A total of 1,106 authors published papers on the topic of *H. pylori* and IBD, 54 of whom have published more than 3 papers on the subject. The clusters of authors are illustrated in Figure 3C. Among them, 11 authors form the most closely collaborative cluster (link strength 3). The top eight authors with the most publications are shown in Table 3. Prof. Kao, John Y. published the most documents (10 records), followed by Prof. Luther, Jay (6 records) and Prof. Zhang, Min (6 records).

**Table 3:** Top 8 most active authors.

No.	Authors	Publications	Citations	Total link strength
1	Kao, John Y.	10	380	23
2	Luther, Jay	6	337	12
3	Zhang, Min	6	197	16
4	Archavlis, E.	5	16	14
5	Mantzaris, G. J.	5	16	14
6	Owyang, Stephanie Y.	5	151	16
7	Petraki, K.	5	16	14
8	Shoenfeld, Yehuda	5	455	0

A total of 112 journals published the pertinent papers, of which 25 journals published more than 5 papers. The two most prolific journals were Gastroenterology (*n* = 24), followed by Helicobacter (*n* = 22), collectively representing 19% of the publications in the WOSCC database. Table 4 presents the top 9 most active journals.

**Table 4:** Top 9 most active journals.

No.	Journals	Publications	Citations	Total link strength	IF (2023)
1	Gastroenterology	24	355	11	29.4
2	Helicobacter	22	410	54	4.4
3	World Journal of Gastroenterology	13	722	63	4.3
4	Inflammatory Bowel Diseases	12	732	57	4.9
5	Gut	11	642	72	24.5
6	Alimentary Pharmacology & Therapeutics	7	220	46	7.6
7	American Journal of Gastroenterology	7	30	8	9.8
8	Journal of Gastroenterology and Hepatology	7	17	7	4.1
9	Scandinavian Journal of Gastroenterology	5	184	34	1.9

### 3.4 Keyword visualization and co-occurrence analysis

**Figure 4:**
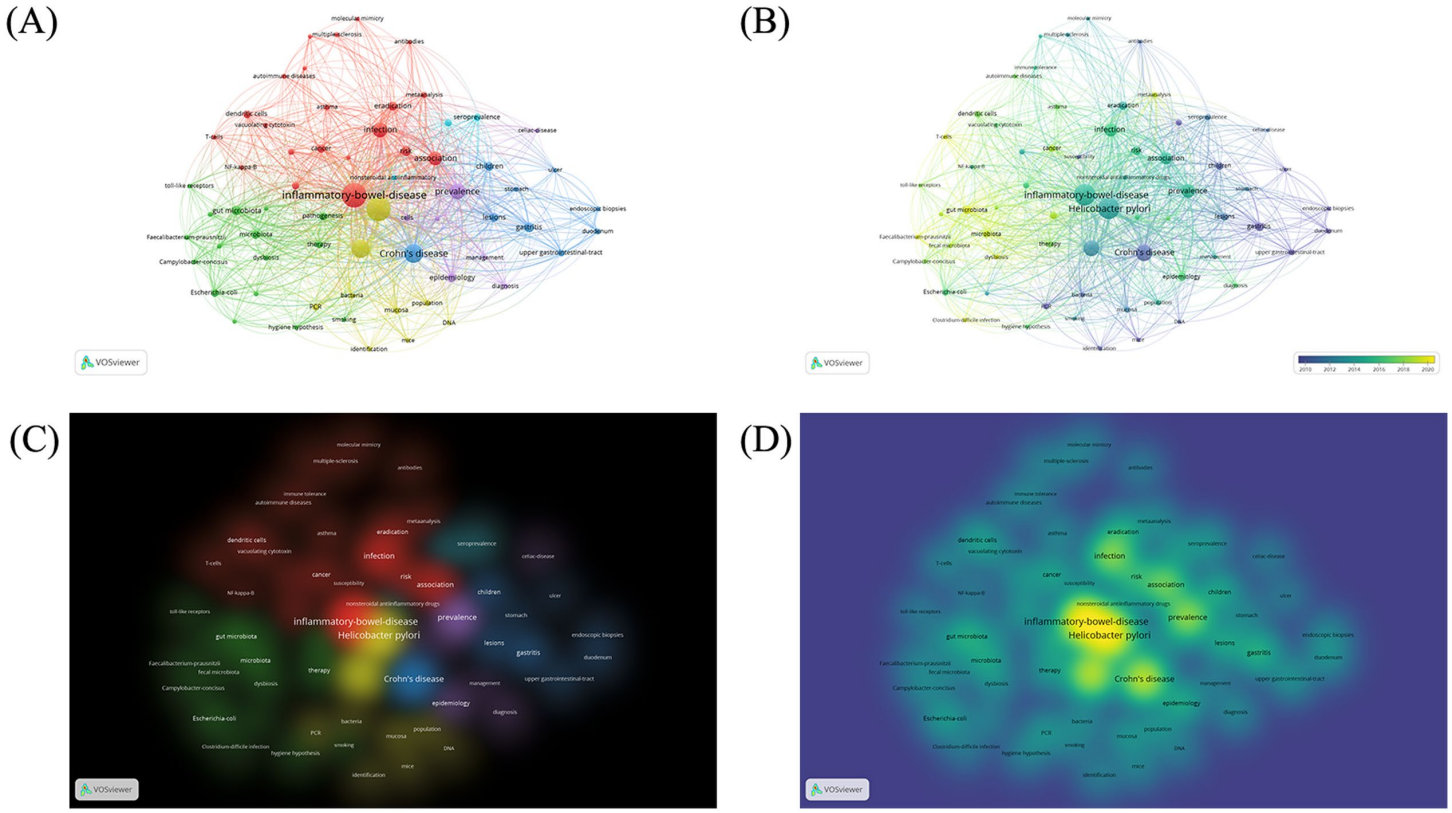
Keyword co-occurrence bibliometric analyses. **(A)** Network visualization. **(B)** Overlay visualization. **(C)** Item density visualization. **(D)** Cluster density visualization.

We manually standardized the keywords, merged and replaced similar keywords before constructing the cluster maps. The analysis involved a total of 65 keywords, selected from 854 keywords in total (occurrence >5). The four most popular keywords were “inflammatory-bowel-disease” (TLS: 795, occurrences: 146), “*Helicobacter pylori*” (TLS: 760, occurrences: 141), “Crohn’s Disease” (TLS: 481, occurrences: 83), and “ulcerative colitis” (TLS: 453, occurrences: 78). The analysis yielded six distinct clusters of keywords (Figure 4). Cluster 1 comprised 21 items that represented diseases and immunity (shown in red) including keywords such as vacuolating cytotoxin, regulatory T-cells, NF-kappa-B, dendritic cells, immune tolerance and molecular mimicry. Cluster 2 included 16 items associated with intestinal microbiota (shown in green) including keywords such as *Campylobacter concisus*, *Escherichia coli*, *Faecalibacterium prausnitzii* and fecal microbiota. Cluster 3 comprised 9 items that represented intestinal symptoms (shown in blue) with keywords including Crohn’s disease, gastritis, lesions and ulcer. Cluster 4 contained 9 items that related to the diagnosis of bacterial infections and experimental techniques (shown in yellow) and included keywords such as DNA, PCR, and Cluster 5 comprised 7 items that described clinical features and status of diseases (shown in purple) and included keywords such as cytokine production, diagnosis and prevalence. Cluster 6 consisted of 3 items pertaining to therapeutic treatments for IBD (shown in orange), including keywords such as nonsteroidal anti-inflammatory drugs, seroprevalence, sulfasalazine.

The time trend analysis revealed that early research concerning the association between *H. pylori* infection and IBD predominantly concentrated on the characterization and diagnosis of clinical features and related symptoms. Currently, there is a switch in focus towards elucidating the mechanisms of *H. pylori* infection on IBD, including gut microbiota and immunological mechanisms.

## 4 Discussion

**Figure 5:**
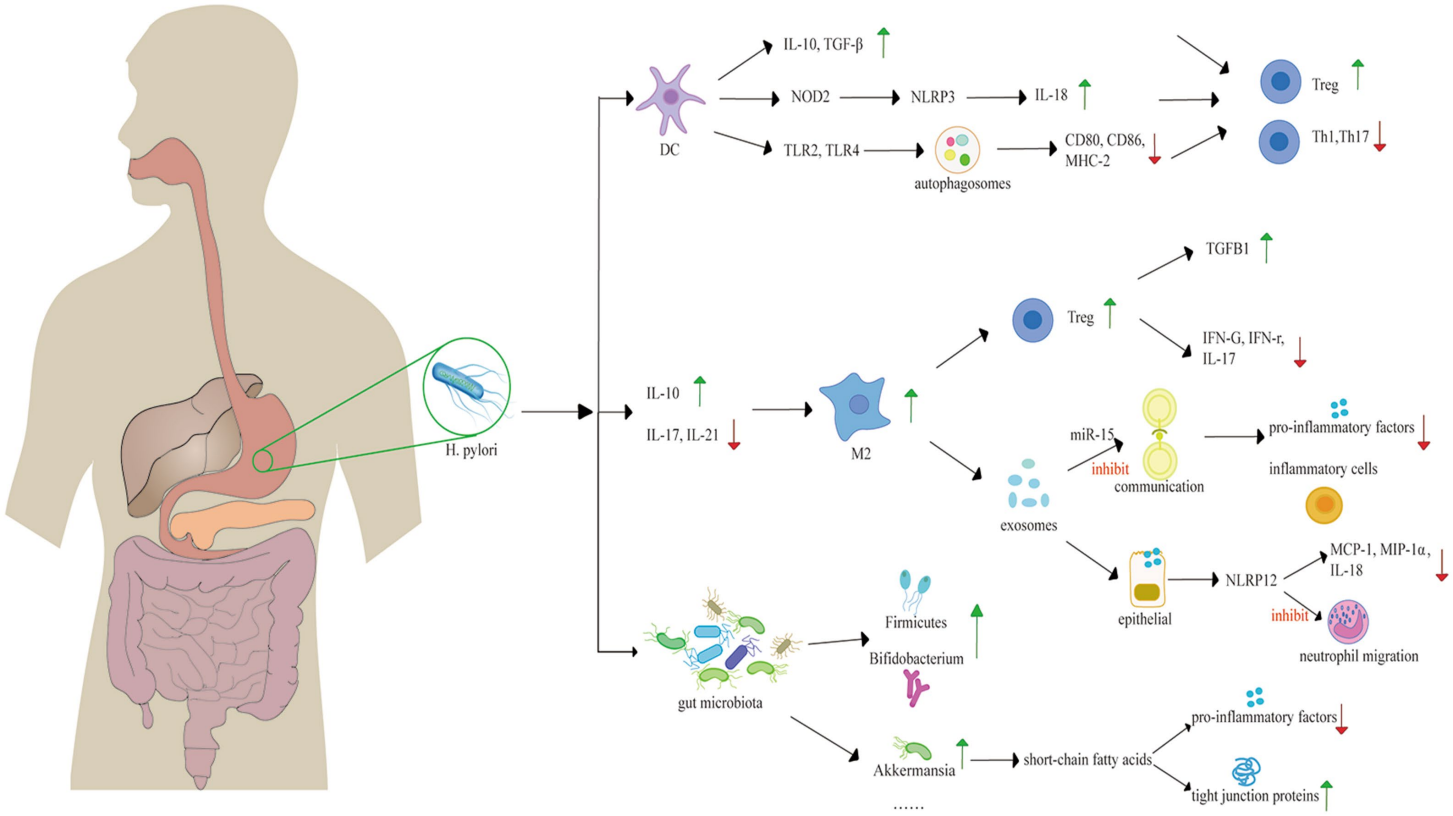
Mechanisms underlying the protective role of *H. pylori* against IBD, including the change of gut microbiota and immune system regulation. *H. pylori* promotes the transformation of DCs into tolerogenic DCs, expressing high levels of anti-inflammatory factors, such as IL-10 and TGF-β. *H. pylori* activates NOD2 to activate the NLRP3 inflammasome of DCs, leading to the expression of IL-18. *H. pylori* triggers autophagosome activation, diminishing the secretion of MHC-2, CD80 and CD86. The secretion of these immune factors induce the differentiation of immunosuppressive Tregs, rather than Th1 or Th17 cells. *H. pylori* also induces the switch of M2 macrophages with increasing IL-10 and diminishing IL-17 and IL-21. M2 macrophages can induce the proliferation of Treg, conferring an anti-inflammatory effect. Exosomes derived from macrophages can suppress the inflammatory response. *H. pylori* alters gut microbiota with a healthier diversity, increasing SCFAs production, and protecting the intestinal barrier with increased tight junction proteins. *H. pylori*, *Helicobacter pylori*; DC, dendritic cell; IL, interleukin; TLR, Toll-like receptor; Treg, regulatory T cell; M2, M2 macrophage.

Utilizing bibliometric analysis, we analyzed 246 articles published from 1994 to February 20, 2024. Most studies find a negative relationship between *H. pylori* infection and IBD. This relationship is usually demonstrated from the following three perspectives: individuals with *H. pylori* infection are less likely develop IBD (Elghannam et al., 2024; Ravikumara, 2023; Kahlam et al., 2023; Kayali et al., 2018); the prevalence of *H. pylori* infection is diminished in patients with IBD (Mizukami et al., 2023); and the eradication of *H. pylori* is associated with an increased risk of IBD (Taskin, 2023). Animal studies demonstrated that colonization by *H. pylori* reduces colonic inflammation and lessens the damage to the colonic mucosal induced by dextran sulfate sodium (DSS) administration (Hu et al., 2024; Kim et al., 2013). However, some researchers posit that there is no significant correlation between *H. pylori* infection and the onset of IBD (Kong et al., 2023; Pokrotnieks and Sitkin, 2023). For example, researchers explain the association between *H. pylori* infection and IBD by the fucosylation status of host proteoglycan in gastrointestinal tract, that is, the two are non-causal (Pokrotnieks and Sitkin, 2023). We summarized the critical publications that elucidate potential mechanisms of the protective effect on IBD in Table 5. The mechanisms of *H. pylori* infection focus on the regulation of immune system (Shirzad-Aski et al., 2021; Setshedi and Watermeyer, 2022; Wang et al., 2022) and alterations in the composition of the intestinal flora when infected (Bai et al., 2022; Liang et al., 2022) (Figure 5).

**Table 5:** Studies about the effects of *H. pylori* on IBD.

No.	Study	Year	Journal	Effect	Mechanism
1	Immune response modulation in inflammatory bowel diseases by *Helicobacter pylori* infection (Balani et al., 2023)	2023	World Journal of Gastroenterology	Protective effect	*H. pylori* infection culminates in high levels of TGF-β and low levels of IL-17 and IL-22 on IBD. It also induces the differentiation of Tregs and the polarization of the M1 macrophage into M2 macrophage lineage. And the ability of *H. pylori* neutrophil-activating protein to reduce Th2 activity may be a possible explanation for the improvement of IBD
2	*Helicobacter pylori* may participate in the development of inflammatory bowel disease by modulating the intestinal microbiota (Bai et al., 2022)	2022	Chinese Medical Journal	Protective effect	*H. pylori* infection increased the diversity of the intestinal microbiota, reduced the abundance of Bacteroidetes, augmented the abundance of Firmicutes, and produced short-chain fatty acid-producing bacteria such as Akkermansia. All these factors may decrease vulnerability to IBD
3	New insights into bacterial mechanisms and potential intestinal epithelial cell therapeutic targets of inflammatory bowel disease (Liang et al., 2022)	2022	Frontiers in Microbiology	Protective effect	The protective effect possibly due to the ability of *H. pylori* to inhibit the growth of other bacteria
4	Association Between *Helicobacter pylori* colonization and inflammatory bowel disease a systematic review and meta-analysis (Shirzad-Aski et al., 2021)	2021	Journal of Clinical Gastroenterology	Protective effect	*H. pylori* stimulated dendritic cells enter a semimature state, leading to the differentiation of immunosuppressive Tregs. Tregs can inhibit the transformation of naive Th0 cells to Th1 and Th17, which play a protective effect on IBD.
5	Serum exosomes derived from Hp-positive gastritis patients inhibit MCP-1 and MIP-1α expression via NLRP12-Notch signaling pathway in intestinal epithelial cells and improve DSS-induced colitis in mice	2020	International Immunopharmacology	Protective effect	Serum exosomes patients with *H. pylori* infection can promote NLRP12 expression in intestinal epithelial cells, and NLRP12 decreased chemokine MCP-1 and MIP-1α expression by inhibiting the Notch signaling pathway, which improved colitis symptoms in DSS-induced colitis mice
6	Environmental risk factors for inflammatory bowel diseases: an umbrella review of meta-analyses (Piovani et al., 2019)	2019	Gastroenterology	Protective effect	*H. pylori* infection may reduce intestinal inflammation through Toll-like receptor 2 and interleukin 10 production, the inhibition of type I interferon and interleukin 12 production, and the accumulation of regulatory T cells
7	The gut microbiota in the pathogenesis and therapeutics of inflammatory bowel disease (Zuo and Ng, 2018)	2018	Frontiers in Microbiology	Protective effect	*H. pylori* infection could induce immune tolerance and limit inflammatory responses
8	Role of the gut microbiota in inflammatory bowel disease pathogenesis: What have we learnt in the past 10 years? (Hold et al., 2014)	2014	World Journal of Gastroenterology	Protective effect	The protective effect may conform to the “hygiene hypothesis” for the development of IBD

### 4.1 *Helicobacter pylori* infection affects intestinal immune mechanisms in patients with IBD

Immunosuppression may explain the negative link between *H. pylori* infection and IBD (Murad, 2016). Tolerogenic *H. pylori*-specific dendritic cells (DCs) are fundamental in immunosuppression. As the most potent antigen-presenting cells and the unique activators of naive T lymphocytes (Th0), DCs play a key role in modulating adaptive immunity through presenting pathogen antigens and inducing the differentiation of Th0 cells into various lymphocyte subsets (Yu et al., 2018). Tolerogenic DCs induced by *H. pylori* express less MHC-2, CD80, CD86, and CD40; secrete fewer pro-inflammatory factors and produce increasing anti-inflammatory factors, such as interleukin (IL)-10, IL-18, and TGF-β (Yu et al., 2018). This process fosters a specific immune microenvironment that mediates the differentiation of immunosuppressive Tregs, rather than T helper 1 cells (Th1) or Th17 cells from naive Th0 cells (Papamichael et al., 2014). Th1 cells and Th17 cells secret interferon (IFN)-γ, IL-17, and IL-23, which protect the body from pathogens. However, excessive activation of Th1 and Th17 cells disrupt the immunological balance between helper T cells and regulatory T cells, leading to cytokine dysregulation, which plays a significant role in the development of IBD (Park et al., 2023; Zhang et al., 2020; Stromnes et al., 2008). Furthermore, Tregs inhibit the conversion of Th0 cells into Th1 and Th17 cells and sustain the tolerogenic state of DCs through direct contact and the secretion of IL-10 and TGF-β (Arnold and Müller, 2016). Tregs in the gastric mucosa of *H. pylori*-infected individuals can migrate to other lymphoid tissues in intestinal tract, exerting an protective effect (Yu et al., 2018).

The induction of tolerogenic DCs by *H. pylori* involves multiple potential mechanisms. One such mechanism contains the NLR family pyrin domain containing 3 (NLRP3) inflammasome. NLRP3 inflammasome is a multiprotein complex that plays a crucial role in the innate immune response (Balani et al., 2023). *H. pylori* binds to pattern recognition receptors-Toll like receptor 2 (TLR2) and nucleotide oligomerization domain 2 (NOD2) presented on DCs, activating the inflammasome, thereby culminating in the cleavage/activation of caspase-1, an enzyme responsible for processing pro-IL-1β and pro-IL-18. IL-18 is related to the differentiation of Tregs and the modulation of immune responses mediated by CD4^+^ T cells (Balani et al., 2023; Engler et al., 2015). Studies demonstrate that the activation of NLRP3 may improve the prognosis of IBDs-mice infected with *H. pylori*, which exhibits less severity of DSS-induced colitis, and characterized by notably reduced inflammation and less epithelial alterations (Kim et al., 2013). Another mechanism involves the formation of autophagosome in DCs induced by *H. pylori* via TLR2/TLR4. CD80, CD86, and MHC-2 molecules accumulate within the autophagic vacuoles that contain *H. pylori*, which down-regulates their surface expression, thereby impairing the function of DC cells (Wang et al., 2010).

The macrophage phenotype switch plays a role in the correlation between *H. pylori* infection and IBD. Macrophages are categorized into M1 and M2 subtypes, according to their polarization state. M1 macrophages are pro-inflammatory cells, contributing to a tissue-destructive pro-inflammatory response, while M2 macrophages exhibit anti-inflammatory properties and participate in tissue repair after injury. The conversion of the M1 macrophage lineage into M2 will lead to milder inflammatory responses (Mills, 2014). Researches demonstrate that colonization of *H. pylori* is associated with the phenotypic switch of M1subtype towards M2, which is linked to concomitant increase of CagA-dependent Th2 pattern cytokines and more Treg cells, and TGF-β, as well as the inhibition of TLR-mediated signaling pathways, resulting in decreased levels of IL-17F and IL-21 (Zhang et al., 2018; Marotti et al., 2008). Experiments show that TGF-β is key in the differentiation of Tregs-inducing by M2 macrophages (Schmidt et al., 2016; Tiemessen et al., 2007). Tregs induced by M2 macrophages reduce expression of pro-inflammatory cytokines, including lower levels of *IFNG* mRNA, IFN-γ, and IL17A mRNA, while express higher level of TGFB1 mRNA. These results suggest that M2-Tregs possess an ability of immunosuppressive (Schmidt et al., 2016).

Exosomes generated from macrophages induced by *H. pylori* play a role in the immunosuppression. Exosomes that contain microRNA-155, serve as molecular carriers in the communication between immune cells. MiR-155 is a negative regulator, diminishing the secretion of pro-inflammatory factors. Murine experiments demonstrate that the presence of miR-155 within exosomes correlates with a reduction of inflammatory cells, such as neutrophils and monocytes (Wang et al., 2016).

Serum exosomes induced by *H. pylori* infection can modulate the immune response. Research indicates that the serum exosomes lead to an upregulation of NLRP12 expression in intestinal epithelial cells, which inhibits the expression of chemokine MCP-1 and MIP-1α in these cells, alleviating DSS-induced colitis (Chen et al., 2020). Similar to NLRP3, NLRP12 activates NLRP12 inflammasomes, which activates caspase-1, regulating the release of interleukin like IL-18 (Wang et al., 2002). The persistent activation and excessive recruitment of neutrophils are characteristic features of various inflammatory diseases (Drury et al., 2021), producing high levels of reactive oxygen species (ROS), proteases, pro-inflammatory cytokines and mediators, such as IL-8, TNF-α, and leukotriene B4, leading to damage of the epithelial barrier and recruit monocytes and more neutrophils to the gastrointestinal tract (Biasi et al., 2013; Wéra et al., 2016; Zhou and Liu, 2017; Aviello and Knaus, 2017). However, NLRP12 deregulates the TLR-mediated expression of inflammatory cytokines (Zaki et al., 2011), modulates neutrophil functions and inhibits their migration (Zamoshnikova et al., 2016).

In conclusion, *H. pylori* infection protects against the development of IBD through interaction with various immune components, including tolerogenic DCs, Tregs, inflammasomes, macrophages, exosomes, and so on. However, this information alone does not comprehensively elucidate the mechanisms underlying the protective effects of *H. pylori*, warranting further investigation.

### 4.2 *Helicobacter pylori* infection alters the gut microbiota in patients with IBD

Dysbiosis of gut microbiota is fundamental pathogenic factor of IBD (Hu et al., 2024). Studies demonstrate that patients with IBD exhibit an altered intestinal microecological environment, characterized by diminished microbial diversity and increased bacterial instability. The microbiota in canines with experimentally induced IBD identifies *Campylobacter* as the predominant genus (26.12%), followed by *Helicobacter* (24.53%), *Anaerobiospirillum* (16.52%), and *Fusobacterium* (10.16%), collectively constituting 77.33% of the bacterial composition (Mao et al., 2023). At the genus level, there are significant increases in *Actinobacteria*, *Proteobacteria*, *Roseisolibacter*, *Rhodoplanes*, *Collinsella*, *Pseudarthrobacter*, *Angelakisella*, and *Campylobacter*, alongside a notable decrease in the relative abundance of *Bacteroidetes*, *Firmicutes*, *Oscillospira*, *Alistipes*, *Barnesiella*, *Gastranaerophilales*, *Allobaculum*, *Subdoligranulum*, *Phascolarctobacterium*, *Parabacteroides*, *Prevotella*, *Alloprevotella*, *Ruminococcus torques* group, *Faecalibacterium*, *Sutterella*, and *Desulfovibrio*, and bacteria producing short-chain fatty acid (SCFA) (Mao et al., 2023; Caruso et al., 2020; Andoh and Nishida, 2023). In individuals infected with *H. pylori*, study indicates a decrease in the levels of *Bacteroidetes* (Fujimori, 2021). However, *H. pylori* infection also results in an increase in the abundance of *Firmicutes*, and probiotic bacteria such as *Bifidobacterium*, *Lactobacillus*, and short-chain fatty acid-producing bacteria including *Akkermansia* and members of the *Muribaculaceae* family within the *Bacteroidetes phylum* (Balani et al., 2023; Bai et al., 2022; Hu et al., 2024). These alterations contribute to a more diverse and healthier alpha diversity of the intestinal microbiota (Hu et al., 2024). Therefore, the alterations in the intestinal flora induced by *H. pylori* may be linked to the protective effects against IBD.

SCFA may be involved in molecular patterns that cause the alteration of microbiota. SCFA may have a protective effect on intestinal tract (Liang et al., 2022). It interacts with G protein-coupled receptors, modulate immune cell activity, and diminish the expression of pro-inflammatory factors (Nicolas and Chang, 2019), influencing immune responses (Vieira et al., 2015). Furthermore, SCFA contributes to the maintenance of intestinal barrier integrity and homeostasis (Vieira et al., 2015). Researches show that in the colon of DSS-treated mice, the expression of three tight junction proteins-claudin 1, occludin, and ZO-1, essential for preserving intestinal barrier function, is reduced. However, this decrease is significantly reversed after the colonization by *H. pylori*, suggesting a function of intestinal barrier repaired by *H. pylori* colonization (Hu et al., 2024; Chelakkot et al., 2018).

Furthermore, several studies suggest that the protective effect of *H. pylori* involves the combination of the secretion of gastric acid (Balani et al., 2023), intestinal hormones (Lopetuso et al., 2014), and the presence of an intact type IV secretion system (T4SS) (Heimesaat et al., 2014). Chronic *H. pylori* infection results in hypochlorhydria, which enables colonization of the distal intestine by acidic pH-sensitive bacteria; *H. pylori* infection also enhance the secretion of gastrin and leptin, modifying gut metabolism (Balani et al., 2023). Additionally, the presence of an intact T4SS in *H. pylori* is linked to alterations in the composition of the microbiota of distal gastrointestinal tract. Strains with a cag pathogenicity island are capable of translocating the effector protein CagA into host cells via the T4SS (Wessler and Backert, 2008). Once inside the host cell, CagA gets tyrosine phosphorylated by host kinases and interferes signal transduction pathways that regulate cell polarity, inflammation, proliferation, and apoptosis (Hu et al., 2024; Heimesaat et al., 2014). Despite the pathogenic potential associated with *H. pylori* CagA^+^, these strains may positively influence the prognosis of IBD. A meta-analysis indicates a significant reduction in the prevalence of IBD associated with *H. pylori* CagA^+^ strains, particularly in CD (Tepler et al., 2019; Bretto et al., 2024). Hypochlorhydria and hypergastrinemia are observed in experiments involving Mongolian gerbils infected with *H. pylori* T4SS^+^, which may be an explanation for the alterations in the microbiota within the gastrointestinal tract (Rieder et al., 2005).

### 4.3 The interaction between *Helicobacter pylori* therapy and treatment of IBD

*H. pylori* infection causes chronic gastritis, which can progress to severe gastroduodenal pathologies, such as peptic ulcers, gastric cancer, and gastric mucosa-associated lymphoid tissue lymphoma (Malfertheiner et al., 2023). Thus, the eradication therapy for *H. pylori* is recommended by all societies (Elghannam et al., 2024). However, researches indicate that patients may be at an increased risk of developing CD or UC after the eradication therapy (Mizukami et al., 2023; Murad, 2016; Tursi, 2006; Jovanovic et al., 2001; Liu et al., 2024). A case study reports that a 34-year-old man experienced rapid onset of CD after *H. pylori* eradication. Potential explanations are the imbalance between the Th1 and Th2 immune patterns, which may contribute to susceptibility to CD. *H. pylori* eradication is proven to reduce Th2 pattern cytokines (notably IL-4, IL-5 and IL-6) while increasing Th1 pattern pro-inflammatory cytokines, which may trigger the onset of CD (Murad, 2016; Papamichael et al., 2014). Another hypothesis posits that the antibiotics used to treat *H. pylori* may disrupt the gut microbiome, leading to decreased diversity, similar to the microbiota composition in patients with IBD (Matsumura et al., 2001; Triantafillidis et al., 2003; Huang et al., 2024). However, studies indicate that the eradication of *H. pylori* is not linked to the development of IBD (Kong et al., 2023; Yang et al., 2024; Dilaghi et al., 2024). While a large amount of studies investigate the implication of *H. pylori* eradication on IBD, seldom research exists concerning the effects of antibiotics utilized for *H. pylori* eradication on patients with IBD. Several investigations report different influences of antibiotics employed in quadruple therapy on the development of IBD, including tetracycline, clarithromycin and metronidazole (Maloy, 2012; Bejcek et al., 2024; Lloyd et al., 2020). One study indicates a positive relationship between the use of oral tetracyclines and the onset of IBD, particularly CD (Maloy, 2012). A retrospective cohort study shows that there is an increased risk of *Clostridioides difficile* infections in patients treated with metronidazole-class antibiotics (Bejcek et al., 2024). Additionally, researches propose that clarithromycin may serve as an anti-inflammatory therapeutic agent for IBD, through a combination of innovative bioinformatic analyses and laboratory experiments. Clarithromycin inhibits LPS-induced NF-κB (p65) DNA binding *in vivo*, alleviates DSS-induced colitis in murine models, and suppresses TNF-induced NF-κB (p65) nuclear localization in human ileal organoids, further study is necessary to confirm its effects in patients (Lloyd et al., 2020).

Some meta-analyses suggest that antibiotic therapy may facilitate remission in patients with active CD and UC (Khan et al., 2011; Fedorak and Ismond, 2016), despite the expansion of antibiotic is linked to an increased risk of IBD (Faye et al., 2023; Piovani et al., 2019; Ananthakrishnan et al., 2018) and the converse results from other studies (Huang et al., 2011; Kronman et al., 2012). The underlying mechanisms may be associated with changes in the gut microbiota-different classes of antibiotics induce different patterns of microbiota alteration because of their spectrum and bacterial target. For instance, macrolides, which inhibit protein synthesis, have active coverage against Gram-positive bacteria, including *Actinobacteria* and *Firmicutes* (Ianiro et al., 2016). Additionally, certain antibiotics have beneficial impacts on gut microbiota by promoting the growth of probiotic bacteria. Such as nitrofurantoin, a broad-spectrum antibiotic, is proven to temporarily increase the population of the beneficial *Bifidobacterium* genus (Vervoort et al., 2015).

The medications used to treat IBD include sulfasalazine, mesalazine, corticosteroids and immunosuppressants. Sulfasalazine and mesalazine are classified as 5-aminosalicylic acids, while corticosteroids and immunosuppressants are categorized as immune system inhibitors. A study demonstrates that IBD treatments, such as anti-TNF-α agents, immunosuppressants, or corticosteroids have no relationship with the prevalence of *H. pylori* infection (Triantafillidis et al., 2014). Additionally, research indicates that there is no significant difference in the prevalence of *H. pylori* infection between these two classes of medications (Zhong et al., 2021).

Regarding the eradication of *H. pylori* infection in children, the latest ESPGHAN/NASPGHAN guidelines recommend a discussion with families concerning the advantages and disadvantages of eradication in children diagnosed with *H. pylori*-associated gastritis and other gastrointestinal diseases (Homan et al., 2024). Our study supports this guideline. Besides, it is important to remember that *H. pylori* has coevolved with humans over thousands of years. Despite more than half of the global population is infected, clinical disease occurs only in a small proportion of the infected individuals (Ravikumara, 2023). It has been estimated that the *H. pylori* infection is associated with gastric and duodenal ulcers in 1–10% of infected patients, gastric carcinoma in 0.1–0.3%, and gastric MALT lymphoma in less than 0.01% (McColl, 2010). Therefore, we propose that *H. pylori* infection should not be eradicated unconditionally in every case, as the bacterium may confer a protective effect in patients with IBD. The purpose of this review is not to dispute the fact that *H. pylori* is a class 1 human carcinogen, but to highlight potential beneficial effects against IBD. Clinicians should carefully assess the risks and benefits of *H. pylori* eradication in patients with IBD and comorbid *H. pylori* infection.

## 5 Conclusion

In this study, we explore research trends and hotspots regarding the relationship between *H. pylori* infection and pathogenesis of IBD using bibliometric analysis. To the best of our knowledge, no bibliometrics studies on this specific topic have been published previously. We objectively assess the contributions of various academic institutions and researchers, finding that the number of papers on *H. pylori* and IBD has increased significantly over the past two decades. China, the United States, and Australia are at the forefront of this field with the most published journals being Gastroenterology and Helicobacter.

The mainstream view is that *H. pylori* infection may have a protective effect in IBD patients. Recent studies have delved into molecular mechanisms, concentrating on immune cells, cytokines, and changes in microbial composition. We review several well-researched mechanisms, such as regulation of immune system and alterations in intestinal flora, and also examine the interaction between *H. pylori* treatment and IBD.

Despite notable progress in the last decade, challenges remain. The exact relationship between *H. pylori* and IBD is still uncertain. Many studies suggest that *H. pylori* infection may reduce the risk and severity of IBD (Balani et al., 2023; Bretto et al., 2024; Wu et al., 2023), but others present different perspectives (Mukherjee et al., 2024; Desmedt et al., 2024).

Future studies should focus on clarifying the relationship between *H. pylori* infection and IBD by investigating mechanisms like changes in gut microbiota and regulation of immune. Such research could provide new insights into the treatment of *H. pylori* infection.

Lastly, our study has some limitations. We relied on the WOSCC database, which, though extensive, excludes publications from other sources that may influence results. Additionally, selection bias and publication bias may affect the analysis due to manual screening process used for paper selection.

## Data Availability

The original contributions presented in the study are included in the article/supplementary material, further inquiries can be directed to the corresponding authors.
